# High Expression of Galectin-3 in Patients with IgG4-Related Disease: A Proteomic Approach

**DOI:** 10.1155/2017/9312142

**Published:** 2017-05-16

**Authors:** Adeeb Salah, Hajime Yoshifuji, Shinji Ito, Koji Kitagori, Kaori Kiso, Norishige Yamada, Toshiki Nakajima, Hironori Haga, Tatsuaki Tsuruyama, Aya Miyagawa-Hayashino

**Affiliations:** ^1^Department of Diagnostic Pathology, Kyoto University Hospital, Kyoto, Japan; ^2^Department of Pathology, Faculty of Medicine and Health Sciences, University of Science and Technology, Sana'a, Yemen; ^3^Department of Rheumatology and Clinical Immunology, Graduate School of Medicine, Kyoto University, Kyoto, Japan; ^4^Bio Frontier Platform, Graduate School of Medicine, Kyoto University, Kyoto, Japan; ^5^Center for Innovation in Immunoregulative Technology and Therapeutics, Graduate School of Medicine, Kyoto University, Kyoto, Japan; ^6^Center for Anatomical, Pathological and Forensic Medical Research, Graduate School of Medicine, Kyoto University, Kyoto, Japan

## Abstract

**Objectives:**

Immunoglobulin G4-related disease (IgG4-RD) is a multiorgan condition manifesting itself in different forms. This study aimed to investigate protein expression profiles and to find the possible biomarker for IgG4-RD by liquid chromatography mass spectrometry (LC-MS) using tissue sections in IgG4-RD patients.

**Methods:**

Protein expression profiles in five IgG4-related pancreatitis and three normal pancreatic samples were compared using LC-MS and were validated by quantitative real-time PCR (qRT-PCR), immunoblotting, and immunohistochemistry. ELISA was employed in the serum of 20 patients with systemic IgG4-RD before and during steroid treatment.

**Results:**

LC-MS indicated that the levels of 17 proteins were significantly higher and 12 others were significantly lower in IgG4-related pancreatitis patients compared to controls. Among these proteins, galectin-3 levels were 13-fold higher in IgG4-related pancreatitis (*P* < 0.01). These results were confirmed by immunoblotting and qRT-PCR. The average number of galectin-3 + cells in various organs of IgG4-RD patients, including salivary glands, lungs, and lymph nodes, was higher than in controls. Galectin-3 was detectable in macrophages, dendritic cells, and stromal myofibroblast-like cells, but not in lymphocytes by immunofluorescence staining. Serum galectin-3 levels were higher in patients with IgG4-RD compared with healthy donors and remained high during steroid therapy.

**Conclusion:**

Galectin-3 was overexpressed in IgG4-RD and the levels were indirectly related to clinical activity.

## 1. Introduction

Immunoglobulin G4-related disease (IgG4-RD) is an autoimmune multiorgan condition, characterized by a dense lymphoplasmacytic infiltration with a high number of IgG4-positive plasma cells and high IgG4 levels in serum [1, 2]. The disease has an indolent course and may only be detected after complications dependent on masses [1, 2]. IgG4 is synthesized and secreted by plasma cells as a part of an immune-protective mechanism and accounts for less than 5% of the total IgG in the serum of healthy individuals. IgG4 is generally considered as an anti-inflammatory immunoglobulin, because the ability to fix complement and bind activating Fc receptors is limited [3]. Thus, it is unclear if this immunoglobulin links to disease pathogenesis [2, 4].

Chronic antigen exposure stimulates IgG4 production, leading to a shift in the IgG4 : IgG1 ratio. The mechanism driving this switch still remains unclear. However, Th2 interleukins such as IL-4, IL-5, and IL-13 can mediate the transition from IgG1 to IgG4 [4, 5]. Th2s in CD4+ T cells and regulatory T cells play a role in the excessive production of IgG4 and stromal fibrosis in IgG4-RD [6–9]. However, more recent studies show an abundance of CD4+ cytotoxic T cells and a paucity of Th2 cells in IgG4-RD [10, 11]. About 50% of IgG4-RD patients have also allergic diseases [2, 12] and these conflicting results may come from IgG4-RD with or without a history of atopy [13]. Nevertheless, the etiology and pathogenesis of IgG4-RD are not well known.

Recent advancements in technical procedures and bioinformatic methods improved the sensitivity of mass spectrometry, making proteomic analysis on formalin-fixed paraffin embedded (FFPE) tissues possible [14]. In this paper, we aimed to investigate protein expression profiles in IgG4-RD and to identify possible biomarkers against this condition using liquid chromatography mass spectrometry (LC-MS).

## 2. Materials and Methods

### 2.1. Liquid Chromatography Mass Spectrometry (LC-MS)

Proteins for LC-MS analysis were extracted from FFPE tissue samples (five IgG4-related pancreatitis and three normal pancreas tissue) using the Liquid Tissue MS Protein Prep kit (Expression Pathology Inc, Rockville, MD, USA) [15]. All cases analyzed for LC-MS were the partial resection for pancreas. For normal pancreas tissues, negative surgical margins taken at resection for pancreatic cancer were selected. Briefly, after deparaffinization, three 0.4 *μ*m thick tissue sections (10 × 10 mm) were dissected using a needle and solubilized in 20 uL of Liquid Tissue buffer and protein digestion was performed with trypsin (Promega Corp, Madison, WI, USA) for 18 h at 37°C. Samples were dried and solubilized in 0.1% formic acid (Wako, Osaka, Japan) and 1 *μ*g aliquots for each sample were separated by nanoflow reversed-phase LC (NanoLC-Ultra 2D-Plus, Eksigent, Dublin, CA, USA) equipped with cHiPLC Nanoflex (Eksigent). Eluted peptides were analyzed by a quadrupole-time-of-flight hybrid mass spectrometer (Triple TOF5600+ system, AB SCIEX, Framingham, MA, USA).

### 2.2. Peptides Identification and Quantification

Mass spectra were searched against the Uniprot/Swissprot human proteomic database (2014-6 release) from the European Bioinformatics Institute using ProteinPilot version 4.5*β* (AB SCIEX). False discovery rates (FDRs) were determined after peptide/protein identification using Proteomic System Performance Evaluation Pipeline provided as a part of ProteinPilot (AB SCIEX). Label-free quantification of peptides was performed using Progenesis QI for Proteomics (Nonlinear Dynamics, Newcastle upon Tyne, UK). Proteins identified by at least two distinct peptides were used for further analysis.

### 2.3. Immunoblotting

Immunoblot analysis was performed for protein extracts from FFPE tissues using the Qproteome FFPE Tissue kit (QIAGEN, Venlo, Netherlands). The antibodies used were anti-galectin-3 (9C4, Abcam, Cambridge, UK, 1 : 1000 dilution) and anti-beta-actin (Abcam, 1 : 1000). Protein bands were visualized with a chemiluminescence substrate (Chemi-Lumi One L, Nacalai Tesque, Kyoto, Japan), and blots were imaged using Ez-Capture MG (Daihan Scientific Co., Ltd., Gangwon-do, South Korea). Bands were analyzed using the CS Analyzer (Atto Corporation, Tokyo, Japan).

### 2.4. Real-Time Quantitative PCR (qRT-PCR)

Total RNA was extracted from FFPE samples using the NucleoSpin total RNA FFPE kit (Macherey-Nagel, Düren, Germany). cDNA synthesis was performed using the ReverTra Ace qPCR RT kit (TOYOBO, Osaka, Japan). Amplifications were carried out in triplicate on a 96-well plate in a 10 *μ*L volume per well using Fast SYBR Green Master Mix (Applied Biosystems, Life Technologies, Carlsbad, CA, USA). These reactions were performed with an Applied Biosystems 7500 Fast real-time PCR machine. Expression was normalized to* GAPDH*, using the 2^−ΔΔCt^ method. The primer sequences were* LGALS3* (forward: 5′-GCCTCGCATGCTGATAACAA-3′, reverse: 5′-CGTGGGTTAAAGTGGAAGGC-3′) and* GAPDH* (forward: 5′-GGTATCGTGGAAGGACTCATGAC-3′, reverse: 5′-ATGCCAGTGAGCTTCCCGTTCAGC-3′).

### 2.5. Immunohistochemistry

Analyzed specimens comprised of FFPE tissues from IgG4-RD patients retrieved from the archive of the department of Diagnostic Pathology, Kyoto University Hospital: pancreas (*n* = 5), bile duct (*n* = 3), retroperitoneum (*n* = 1), aorta (*n* = 1), kidney (*n* = 2), salivary gland (*n* = 6), lung (*n* = 4), ureter (*n* = 1), and lymph node (*n* = 6) from 29 patients between January 2007 and August 2015. Paraffin embedded sections from IgG4-RD patients were immunostained with the following antibodies: alpha-smooth muscle actin (1A4, Sigma Aldrich, St. Louis, MO, USA, 1 : 300), CD3 (F7.2.38, DakoCytomation, Glostrup, Denmark, 1 : 50), CD11c (5D11, Cell marque, Rocklin, CA, 1 : 50), CD123 (6H6, eBioscience, San Diego, CA, USA, 1 : 100), CD20 (Clone L26, mouse monoclonal, DakoCytomation, 1 : 1), CD68 (PGM1, DakoCytomation, 1 : 10), galectin-3 (1 : 100), IgG (EPR4421, Abcam, 1 : 50), and IgG4 (HP6025, Nichirei Biosciences Inc., Tokyo, Japan, 1 : 1). The REAL™ EnVision™/HRP detection system (DakoCytomation, Glostrup, Denmark) was used to detect immunohistochemical signals. Double immunofluorescence staining was performed using the TSA Plus Fluorescence kit (PerkinElmer Inc., Waltham, MA, USA).

### 2.6. Enzyme-Linked Immunosorbent Assay (ELISA)

ELISA was used to measure galectin-3 and IgG4 levels in 37 serum samples from 20 patients with systemic IgG4-RD before and during prednisolone (PSL) treatment (pretreatment sera were available from 11 patients) and from five healthy control individuals (Human galectin-3 Quantikine ELISA Kit, R&D Systems, Inc, Minneapolis, MN, USA, and Human IgG subclass profile, Novex, Life Technologies, Frederick, MD, USA, resp.). All experiments were performed in duplicate. Results were compared with samples of patients treated with a tapering dose of PSL.

### 2.7. Patient Consent and Confidentiality

Sample collection and use of clinical records were authorized under the written consent of patients, and the study was conducted according to the principles expressed in the Declaration of Helsinki. The Ethics Committee of Kyoto University approved this study (R0305).

### 2.8. Statistical Analysis

Data were analyzed with GraphPad Prism 6 (mdf, Tokyo, Japan) or R version 3.2.0. To compare continuous variables, unpaired Student's *t*-test or Mann–Whitney *U* test and two-way analysis of variance (ANOVA) followed by Bonferroni's multiple comparisons test were used. Differences with a *P* value smaller than 0.05 were considered statistically significant.

## 3. Results

### 3.1. Protein Identification and Quantification

**Figure 1:**
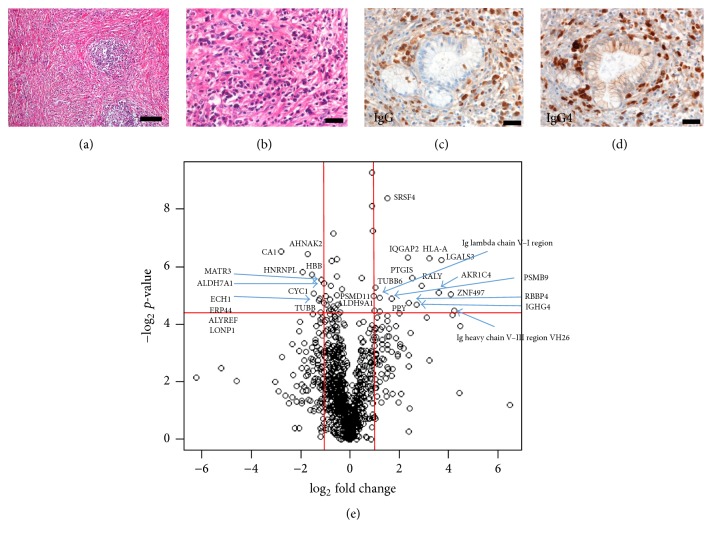
*Histologic features of IgG4-related disease (IgG4-RD) and liquid chromatography mass spectrometry (LS-MS) analysis*. ((a) to (d)) Pancreatic tissue from patients with IgG4-related pancreatitis. (a) Fibrosis and aggregates of lymphocytes. Hematoxylin and eosin staining, scale bar, 100 *μ*m. (b) Plasma cell infiltration as one of the histological features of IgG4-RD, Hematoxylin and eosins staining, scale bar, 20 *μ*m. (c) Immunostaining with anti-IgG antibodies; or (d) with anti-IgG4 antibodies staining. IgG4/IgG > 90%, scale bar, 20 *μ*m. IgG4 staining is one of the diagnostic criteria of IgG4-RD. (e) Volcano plot of all differential proteins showing a different abundance in IgG4-related pancreatitis (*n* = 5) compared to the healthy pancreas (*n* = 3) samples in our LC-MS analysis. Horizontal axis represents fold change compared to the respective protein in control samples. Vertical axis represents *P* values. Proteins with a 2-fold change in their levels and a *P* value lower that 0.05 were considered significant hits. Fold change values indicate higher (+) or lower (−) expression in IgG4-related pancreatitis samples compared with the controls. Significant proteins are labeled with their gene name in the figure.

LC-MS revealed that 956 proteins were differentially expressed between IgG4-related pancreatitis and normal pancreatic samples (Figures 1(a), 1(b), 1(c), and 1(d)). Among these, 352 were significantly upregulated and 604 were downregulated in IgG4-related pancreatitis samples compared with normal pancreatic tissues (Figure 1(e)). Proteins with high confidence values (*P* value < 0.05) and showing a twofold difference in their expression between IgG4-related pancreatitis and normal pancreatic samples were selected for further analysis; the levels of 17 proteins were higher and those of 12 proteins were lower in IgG4-related pancreatitis tissues than in healthy pancreas (Table 1). We selected galectin-3, as it was one of the highly upregulated proteins in IgG4-related pancreatitis (13-fold, *P* value 0.013).

### 3.2. Validation of Proteomic Data by qRT-PCR, Immunoblotting, and Immunohistochemistry

**Figure 2:**
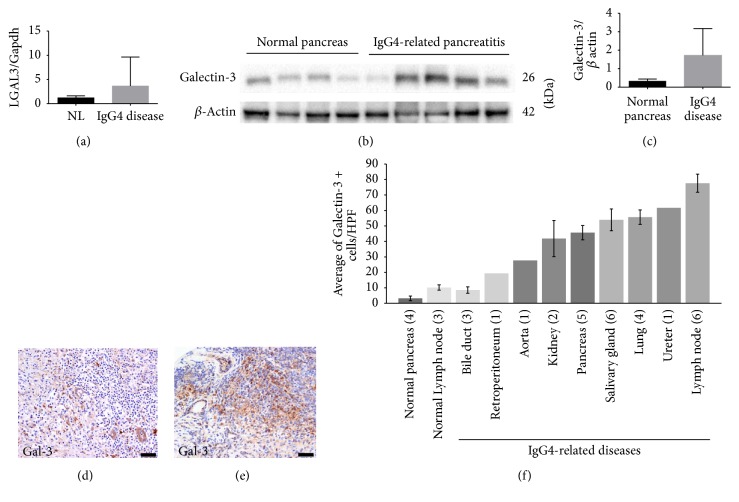
*Validation of galectin-3 expression in patients' tissues*. (a)* LGALS-3* expression was higher in IgG4-related pancreatitis patients compared to controls. Data are presented as the mean relative expression ratio between the mRNA levels of the* LGALS3* gene and the expression of* GAPDH*. Error bars, standard error of the mean (SEM). (b) Immunoblot analysis showing higher galectin-3 protein levels in IgG4-related pancreatitis samples (*n* = 5) compared with those in the normal pancreas samples (*n* = 4). (c) Quantification of the immunoblot presented in panel (b). Mean band intensity ratio was measured as intensity of galectin-3 band divided by the intensity of the corresponding beta actin band. Bar graphs represent mean values. Error bars, SEM. (d, e) Immunolocalization of galectin-3 in IgG4-RD samples ((d) pancreas; (e) submandibular gland). Note that lymphoid cells in (d) were negative for galectin-3. Scale bar, 20 *μ*m. (f) Average galectin-3 positive cells in the stroma in different organs in IgG4-RD patients. Positive cells were counted in 3HPF. Numbers in parentheses represent the number of cases. Bar graphs represent mean values. Error bars, SEM.

IgG4-related pancreatitis samples (*n* = 5) showed a 3-fold up-regulation of* LGALS-3* expression compared with healthy pancreas (*n* = 4; Figure 2(a)). Immunoblotting showed that galectin-3 protein levels in the same samples as qRT-PCR were 5.1-fold higher in IgG4-related pancreatitis samples than that of normal pancreas (Figures 2(b) and 2(c)).

Immunohistochemical analysis showed that galectin-3 mostly stained stromal spindle cells, macrophages-like cells, and epithelial cells, but not mononuclear lymphocyte-like cells (Figures 2(d) and 2(e)).

### 3.3. Galectin-3 Is Highly Expressed in Different Organs with IgG4-RD

To measure the expression of galectin-3 in different organs of IgG4-RD patients, FFPE samples from various organs were stained with anti-galectin-3 antibodies. Since we focused on immune cells, but not on epithelial cells, we counted galectin-3-positive cells in the stroma, including immune and fibroblast-like cells but excluding galectin-3-positive epithelial cells, in high-power fields (HPF) from each specimen. The average number of galectin-3-positive stromal cells was higher in organ samples from IgG4-RD patients compared with healthy pancreas or lymph node samples. The differences between healthy pancreas and IgG4-related pancreatitis samples and between healthy lymph node and IgG4 lymphadenopathy were statistically significant (*P* value < 0.001 for both comparisons) (Figure 2(f)).

### 3.4. Galectin-3 Is Expressed in Macrophages, Dendritic Cells, and Myofibroblasts

**Figure 3:**
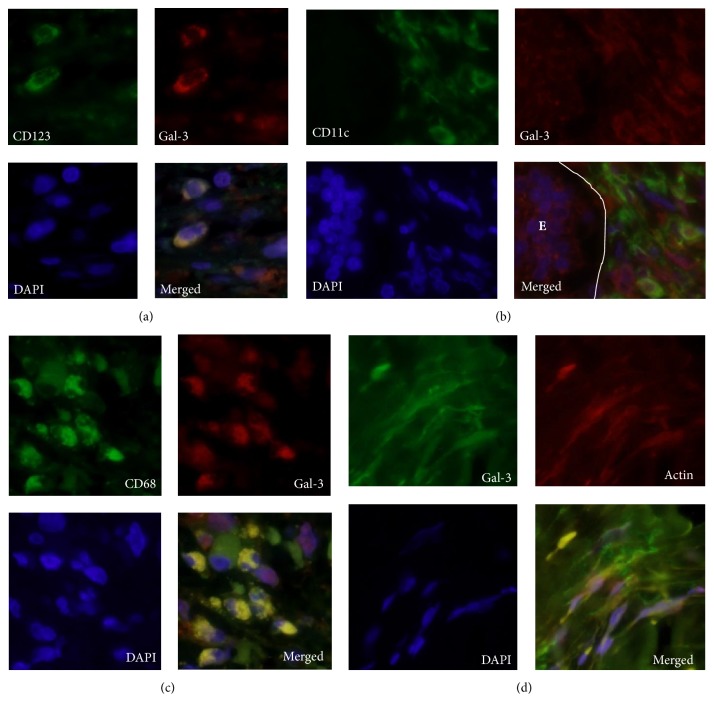
*Immunofluorescent localization galectin-3 in immune and stromal cells of IgG4-RD patients*. (a) Immunofluorescent localization of CD123 (green) and galectin 3 (red) in IgG4-RD samples, showing galectin-3 expression in plasmacytoid dendritic cells. (b) Immunofluorescent localization of CD11c (green) and galectin 3 (red) in IgG4-RD samples, showing galectin-3 expression on myeloid dendritic cells. Note the epithelial cells in the duct, on the left (E), positive for galectin-3. (c) CD68 (green) and galectin-3 (red) in IgG4-RD samples, showing galectin-3 expression on macrophages. (d) Galectin-3 (green) and *α*-smooth muscle actin (red) localization in IgG4-RD samples. Stromal spindle cells were positive for galectin-3, suggesting galectin-3 expression in myofibroblasts. Note the galectin-3 deposition in the stroma. In all experiments, nuclei were stained with DAPI (blue) and images were taken at a magnification of 600x. Gal-3, Galectin-3.

Immunofluorescence staining showed that galectin-3 colocalized with CD68, CD11c, and CD123, suggesting that galectin-3 was expressed in macrophage, myeloid, and plasmacytoid dendritic cells (Figures 3(a), 3(b), and 3(c)). Notably, stromal myofibroblast-like spindle cells expressed both alpha-smooth muscle actin and galectin-3 (Figure 3(d)). Galectin-3 was also visible in the stroma, while we found no galectin-3 staining in CD3-positive T cells or CD20-positive B cells.

### 3.5. Galectin-3 Levels in Serum of Patients with Systemic IgG4-RD

**Figure 4:**
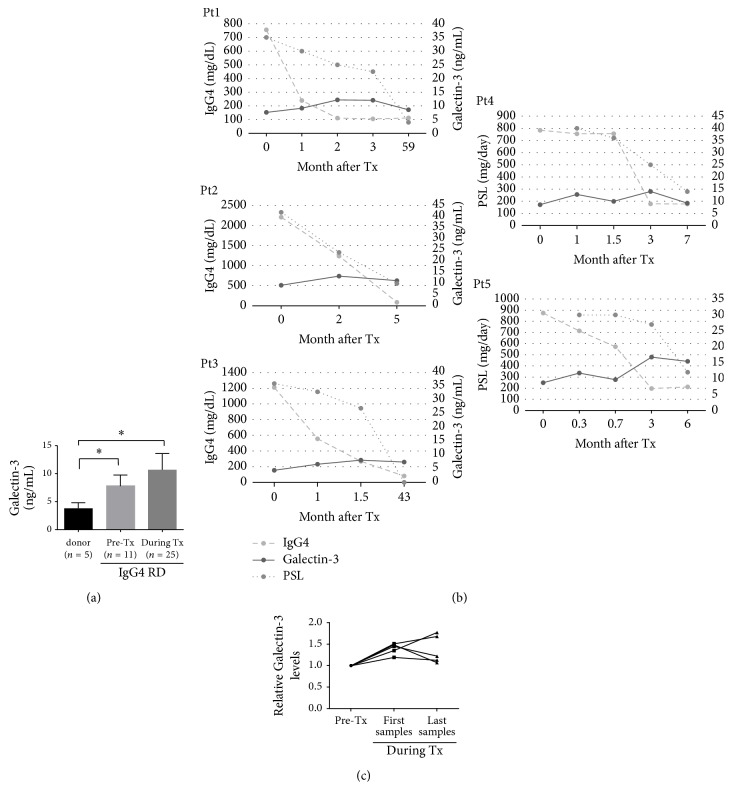
*IgG4 and galectin-3 levels in serum after steroid treatment*. (a) ELISA assay indicates that galectin-3 levels in serum in patients with IgG4-RD were 2- and 2.5-fold higher, before and after steroid treatment, respectively, compared to the normal control (^*∗*^*P* < 0.05). Error bars, standard error of the mean (SEM). Tx, treatment. (b) Changes in IgG4 and galectin-3 levels in serum of five IgG4-RD patients before and during steroid treatment. (c) Galectin-3 levels in serum in five steroid-treated IgG4-RD patients. Galectin-3 levels were 1.39-fold higher immediately after the start of the treatment (mean 1 month) and 1.37-fold higher at the last follow-up treatment after 24 months on average, compared to the pretreatment levels.

Galectin-3 levels in serum of IgG4-RD patients were 7.8 ± 0.58 ng/mL and 10.7 ± 0.58 ng/mL before and during PSL treatment, respectively, that is, 2- and 2.5-fold higher than 3.8 ± 0.46 ng/mL found in healthy donors (versus donor *P* value < 0.01 for both comparisons; Figure 4(a)). We monitored the serum levels of IgG4 and galectin-3 during PSL tapering in five patients for an average of 24 months. While IgG4 serum levels markedly decreased after prednisolone (PSL) therapy, galectin-3 levels in the serum increased. Inversely, lower PSL doses were accompanied by a slight decrease in galectin-3 levels in serum (Figures 4(a) and 4(b)).

Figure 4(c) shows the relative change in galectin-3 levels for five patients before (Galectin-3 levels set to 1) and after treatment (first samples from an average of 1 month after treatment; range 0.3–2 months; last samples from an average of 24 months after treatment; range, 5–59 months).

Galectin-3 levels in serum were on average 1.39 ± 0.058-fold higher immediately after the start of the PSL therapy (on average 1 month) when compared to galectin-3 levels before the treatment. Galectin-3 levels in serum were on average 1.37 ± 0.15-fold higher after the last follow-up treatment, on average 24 months after the start of the therapy, compared to relative to the levels before the treatment (Figure 4(c)).

## 4. Discussion

We chose the protein galectin-3, identified by LC-MS with high confidence and differential expression in FFPE samples of IgG4-related pancreatitis tissues. Galectin-3, encoded by* LGALS3*, is a member of the galectin family and expressed in numerous cells, including epithelium cells of gastrointestinal and respiratory tracts and renal tubules, fibroblasts, chondrocytes, osteocytes, and endothelial cells. This protein has pro- and antiapoptotic activity [16–18] and regulates human monocyte and macrophage, participating in the autophagy pathway [19–21]. Galectin-3 is also expressed in cells involved in immune responses, such as neutrophils, eosinophils, basophils, mast cells, Langerhans cells, dendritic cells, and monocytes and macrophages [18]. Notably, in resting lymphocytes and several lymphoid cell lines, galectin-3 is not normally expressed, but it can be induced by various stimuli [18, 22, 23]. High galectin-3 expression is observed in many forms of cancer, including thyroid, pancreatic, and colon cancers, and has been linked to cancer progression and metastasis [24, 25].

Galectin-3 is highly expressed in tissues affected by several autoimmune diseases, such as systemic lupus erythematosus, polymyositis, dermatomyositis, rheumatoid arthritis, Behcet's disease, systemic sclerosis, and Crohn's disease. Moreover, galectin-3 shows higher expression in active than in inactive diseases [26–28]. Galectin-3 participates in the development of the T helper-2 (Th2) response [29]. However, the expression and the role of galectin-3 in IgG4-RD remains unknown.

This study demonstrated an increase in galectin-3 expression in IgG4-RD patients. Galectin-3 was expressed in cells involved in immune response activity, including macrophages, dendritic cells, myofibroblasts, and epithelial cells. Galectin-3 signal was found in stromal deposition. Dendritic cells are specialized in antigen presentation cells and connect the innate and adaptive immune system [30]. They have the distinctive ability to stimulate and polarize naive T cells. For example, the expressions of IL-12 and IL-10 stimulate T helper-1 (Th1) and Th2 activation, respectively [30]. Th2 response controls the production of IgG4 [18]. Plasmacytoid dendritic cells participate in autoimmune diseases, such as IgG4-related pancreatitis, lupus erythematosus, psoriasis, and contact dermatitis, likely contributing to IFN-*α* production [31, 32]. Galectin-3 secreted by dendritic cells mediates the binding of these cells to lymphocytes and might play a role in cell-cell interactions [33, 34]. Galectin-3 binds integrins, the main membrane molecules involved in cell adhesion, including those present on the surface of macrophages such as *α*1*β*1 integrins and the *α* subunit of *α*M*β*1 integrin (CD11b/18, Mac-1 Ag), and these integrins are used as receptors [18, 21]. Galectin-3 is also essential for effective phagocytosis by macrophages to remove apoptotic cells and therefore preventing autoimmune reactions [35]. In addition, endogenous galectin-3 has been found to drive a Th2 response in both dendritic cells and T cells, while galectin-3 deficiency resulted in the development of a Th1 response [29]. Recent studies show clonally expanded CD4+ cytotoxic T cells that express granzyme B, perforin, IFN-gamma, and TGF-beta1 are prominent in IgG4RD [10, 11] Rituximab-mediated B cell depletion is associated with a reduction in numbers of disease-associated CD4+ cytotoxic T cells, suggesting that B cells play an important role in the maintenance of disease-associated T cell clone [10]. Extracellular galectin-3 directly induces T cell apoptosis in a carbohydrate dependent manner by binding to its cell surface receptors, CD7 and CD29 [36]. Inhibition of galectin-3 in vivo skewed the balance toward plasma cell differentiation and increased immunoglobulin production through the up-regulation of Blimp-1, a transcription factor responsible for B cell apoptosis and essential for plasma cell commitment, in* Trypanosoma cruzi* infection model [23]. Additionally, galectin-3 acts as a negative regulator of the differentiation of B-1 lymphocytes into plasma cells [37]. Likewise, IL-4-mediated B cells activation, resulting in their differentiation into memory cells, is accompanied by a significant increase in galectin-3 expression [23]. Therefore, galectin-3 may act as a protective factor against the progression of IgG4-RD since galectin-3 blocks plasma cell differentiation in B cells and apoptosis of T cells [23, 36, 37], although this protein has other biological roles [18].

In our study, galectin-3 serum levels in patients with IgG4-RD before and during PSL treatment were significantly higher compared with those in healthy donors. IgG4 levels in serum decreased dramatically after PSL treatment, while galectin-3 levels in serum remained high throughout the treatment. Galectin-3 serum levels increased with PSL treatment and gradually decreased with the tapering of PSL. This implies that galectin-3 cannot serve as a realistic biomarker for IgG4-RD in routine clinical practice. Glucocorticoid therapy could be a bias, because galectin-3 expression increases significantly in macrophages and lung Clara cells after glucocorticoid treatment [38]. Similarly, mineralocorticoid hormones increase galectin-3 expression in vascular smooth muscle cells [39]. Therefore, the induction of galectin-3 expression after treatment with PSL, a synthetic glucocorticoid, may be in agreement with these previous reports. Galectin-3 expression is higher in SLE and systemic sclerosis patients with active diseases than those with an inactive one. However, galectin-3 expression is not directly correlated with the disease activity and severity indexes in each SLE and systemic sclerosis patient, nor with the duration of the disease [28]. In our study, galectin-3 was present in the stroma and myofibroblasts in IgG4-RD patients. Immune cells subside immediately after steroid therapy, while tissue fibrosis may remain even after treatment [40]. It is difficult to assist tissue fibrosis because follow-up biopsy is uncommon after the treatment of IgG4-RD. The only available data come from a recent work by ‎Della-Torre et al. [41], who found a reduction in the total ‎number of myofibroblasts after rituximab treatment in one patient with ‎cutaneous IgG4-RD. The presence of extracellular galectin-3 can be a determining factor for prolonged galectin-3 levels in serum after PSL treatment. It is possible that galectin-3 seen in patients with IgG4-RD represents the response against inflammation. To understand the significance of galectin-3 in relation to the pathogenesis of IgG4-RD, the decreased expression of galectin-3 in the affected tissues after successful corticosteroid therapy would be important; however no tissues after therapy were available.

In conclusion, galectin-3 was overexpressed in IgG4-RD and the levels were indirectly related to clinical activity and treatment. Further studies will need to explore the exact mechanism behind the function of galectin-3 in IgG4-RD and other autoimmune diseases.

## Figures and Tables

**Table 1:** Expression profiles of the most significant proteins identified by liquid chromatography mass spectrometry.

	Protein name	Gene name	Location	Function	*P*-ANOVA	Fold change
1	Serine/arginine-rich splicing factor 4	*SRSF4*	Nucleus	Plays a role in alternative splice site selection in pre-mRNA splicing	0.003	2.84
2	Carbonic anhydrase 1	*CA*	Cytoplasm	Reversible hydration of carbon dioxide	0.011	−6.87
3	Protein AHNAK2	*AHNAK2*	Nucleus	Associate with calcium channel proteins in cardiomyocytes	0.011	−3.23
4	Ras GTPase-activating-like protein IQGAP2	*IQGAP2*	Cytoplasm	Interacts with calmodulin and Rho family GTPases	0.012	5.07
5	HLA class I histocompatibility antigen, A-74 alpha chain	*HLA-A*	Plasma membrane	Involved in the presentation of foreign antigens to the immune system	0.013	9.43
6	Galectin-3	*LGALS3*	Cytoplasm, Nucleus	Galactose-specific lectin involved in acute inflammatory responses	0.013	13.01
7	Heterogeneous nuclear ribonucleoprotein L	*HNRNPL*	Cytoplasm, nucleus	Acts as either an activator or repressor of exon inclusion	0.018	−3.80
8	Hemoglobin subunit beta	*HBB*	Red blood cells	Involved in oxygen transportation from the lung to peripheral tissues	0.019	−2.89
9	Prostacyclin synthase	*PTGIS*	Endoplasmic reticulum membrane	Catalyzes the isomerization of prostaglandin H2 to prostacyclin	0.021	5.76
10	Matrin-3	*MATR3*	Nucleus	Role in transcription or may form the internal fibrogranular network	0.022	−2.22
11	Alpha-aminoadipic semialdehyde dehydrogenase	*ALDH7A1*	Cytoplasm	An important cellular osmolyte and methyl donor	0.023	−2.06
12	RNA-binding protein Raly	*RALY*	Nucleus	May be involved in pre-mRNA splicing	0.025	7.44
13	Tubulin beta-6 chain	*TUBB6*	Cytoplasm	A major constituent of microtubules	0.026	2.04
14	Aldo-keto reductase family 1 member C4	*AKR1C4*	Cytoplasm	Catalyzes the transformation of the androgen DHT into the less active form	0.030	12.08
15	Cytochrome c1, heme protein, mitochondrial	*CYC1*	Mitochondria	Transfer electrons to cytochrome C in the mitochondrial respiratory chain	0.030	−2.77
16	Zinc finger protein 497	*ZNF497*	Nucleus	May be involved in transcriptional regulation	0.031	16.87
17	Ig Lambda chainV-I region WAH	NA	Secreted	Antigen binding	0.033	2.29
18	Proteasome subunit beta type-9	*PSMB9*	Cytoplasm, Nucleus	A multicatalytic proteinase complex	0.034	3.21
19	Delta (3,5)-Delta (2,4)-dienoyl-CoA isomerase, mitochondrial	*ECH1*	Mitochondria	Involved in the pathway fatty acid beta-oxidation	0.034	−2.31
20	Endoplasmic reticulum resident protein 44	*ERP44*	Endoplasmic reticulum	Putative role in the control of oxidative protein folding	0.035	−2.38
21	Histone-binding protein RBBP4	*RBBP4*	Nucleus	Putative target chromatin assembly factors	0.037	5.21
22	THO complex subunit 4	*ALYREF*	Cytoplasm, nucleus	Export adapter involved in nuclear export of spliced and un spliced mRNA	0.037	−2.07
23	Ig gamma-4 chain C region	*IGHG4*	Secreted	Constant region of IgG4 heavy chain	0.039	6.47
24	Ig heavy chain V-III region VH26	NA	Secreted	Antigen binding	0.045	18.88
25	26S proteasome non-ATPase regulatory subunit 11	*PSMD11*	Cytoplasm, nucleus	Involved in the ATP-dependent degradation of ubiquitinated proteins	0.045	2.01
26	4-trimethylaminobutyraldehyde dehydrogenase	*ALDH9A1*	Cytoplasm	Catalyzes the irreversible oxidation of a broad range of aldehydes	0.046	2.28
27	Lon protease homolog, mitochondrial	*LONP1*	Mitochondria	Participates in the regulation of mitochondrial gene expression	0.047	−2.26
28	Pancreatic prohormone	*PPY*	Secreted	Acts as a regulator of gastrointestinal functions	0.048	4.01
29	Tubulin beta chain	*TUBB*	Cytoplasm	A major constituent of microtubules	0.049	−2.80

DHT: dihydrotestosterone; ER: endoplasmic reticulum; NA: not available.

Protein with a 2-fold change and *P* < 0.05 (Mann–Whitney *U* test followed by Bonferroni's correction) were considered significant. Fold-change values indicate higher (+) or lower (−) expression in IgG4-related pancreatitis samples compared with controls.

Uniprot/Swissprot human proteomic database as reference.
